# Effectiveness of non-pharmacological therapies on cognitive function in patients with dementia—A network meta-analysis of randomized controlled trials

**DOI:** 10.3389/fnagi.2023.1131744

**Published:** 2023-03-02

**Authors:** Guangxin Luo, Junqiu Zhang, Zeyi Song, Ying Wang, Xiaojing Wang, Haifeng Qu, Fang Wang, Chengjiang Liu, Fujia Gao

**Affiliations:** ^1^School of Public Health, North China University of Science and Technology, Tangshan, China; ^2^School of Clinical Medicine, North China University of Science and Technology, Tangshan, China; ^3^Department of Psychology, The Fourth People’s Hospital of Wuhu, Wuhu, China; ^4^Department of General Medicine, Affiliated Anqing First People’s Hospital of Anhui Medical University, Anqing, China; ^5^Hebei Province Key Laboratory of Occupational Health and Safety for Coal Industry, School of Public Health, North China University of Science and Technology, Tangshan, China

**Keywords:** dementia, cognitive, non-pharmacological therapy, network meta-analysis, randomized controlled trials

## Abstract

**Objective:**

Non-pharmacological therapies (NPTs) have received increasing attention from researchers as a category of treatment to improve cognitive impairment in patients with dementia because of their fewer side effects. In this study, photobiomodulation (PBM), enriched environment (EE), exercise therapy (ET), computerized cognitive training (CCT), and cognitive stimulation therapy (CST) were selected to compare the effects of NPTs that improve dementia by quantifying information from randomized controlled trials (RCTs).

**Methods:**

We did a systematic review and network meta-analysis. We searched PubMed, Embase, Cochrane Central Register of Controlled Trials (CENTRAL), China National Knowledge Infrastructure Database, Wan Fang Database, Chinese Biomedical Literature Database, Web of Science, and VIP Database from the time of database creation to 1 August 2022. Two investigators independently screened the literature, extracted information, and assessed the RCTs’ quality with the Cochrane Collaboration Network Risk of Bias 2.0. Network meta-analysis was performed using R language (X64 version 4.1.3) and STATA 17.0.

**Results:**

We identified 1,268 citations and of these included 38 trials comprising 3,412 participants. For improving dementia, the results of the network meta-analysis showed that compared with the control group (CON), PBM (SMD = 0.90, 95% CI: 0.43–1.37), EE (SMD = 0.71, 95% CI: 0.02–1.41), ET (SMD = 0.42, 95% CI: 0.16–0.68), and CST (SMD = 0.36, 95% CI: 0.11–0.62) were significantly different (*P* < 0.05); There was no significant difference in CCT (SMD = 0.41, 95% CI: −0.07–0.88) (*P* > 0.05). The ranked results showed that PBM has more potential to be the best intervention (*P* = 0.90). In addition, there was a significant difference between PBM and CST in improving cognitive function (SMD = 0.54, 95% CI: 0.00; 1.08, *P* < 0.05).

**Conclusion:**

In this study, NPTs have excellent potential to improve cognition in people with dementia, and PBM may have more significant benefits in improving cognition than the other four NPTs.

**Systematic review registration:**

https://www.crd.york.ac.uk/prospero/, identifier CRD42022363746.

## 1. Introduction

Dementia, a common neurodegenerative disease, was getting more and more attention with the progress of the global population aging. According to the World Health Organization (WHO), there were approximately 55 million cases of dementia patients worldwide, and the number of dementia patients will continue to rise as the world population ages. It is estimated that by 2,050, the number of dementia patients will increase to 139 million (Serge Gauthier et al., 2022). In addition, the total estimated cost of dementia was $1.3 trillion by 2020, which was set to rise with dementia patients in 2030 (World Health Organization, 2021). These ever-increasing patients have brought a double burden on society and economy, while the increase of the estimated prevalence and incidence of dementia emphasized the necessity of effective treatment.

At present, there was a controversy about the pathogenesis of dementia, which had led to the failure to make breakthroughs in drug research on the etiological treatment at this stage. The current drug treatment mainly included two types of cholinesterase inhibitors and ionotropic glutamate receptor antagonists (Tisher and Salardini, 2019), which may cause side effects such as gastrointestinal discomfort, constipation, syncope, falls, arrhythmias, and extrapyramidal symptoms (Cummings et al., 2019), although they had specific improvement effects on patients’ clinical manifestations. In contrast, non-pharmacological therapies (NPTs), which aimed at improving dementia in the elderly, had attracted considerable attention due to their safe, relatively inexpensive, and scalable intervention. At present, the routine NPTS research on dementia patients showed that exercise therapy (ET), cognitive stimulation therapy (CST), and computerized cognitive training (CCT) indicate better treatment effects (Liang et al., 2019). And enriched environment (EE) and photobiomodulation (PBM), as new treatment modalities, have shown sound therapeutic effects in recent studies (Bourdon and Belmin, 2021; Salehpour et al., 2021). However, the efficacy of NPTs were controversial, because the results of individual studies vary widely depending on the training contents. Meanwhile, most of the above NPTs in previous studies have focused on comparing the effectiveness of single non-pharmacological intervention with conventional care in reducing cognitive impairment in patients with dementia. The lack of direct comparative studies of different NPTs leads to differences on which non-pharmacological interventions are most effective (Sikkes et al., 2021).

To tackle this problem, a network meta-analysis is well suited, because it facilitates comparisons of multiple pairs of interventions in one statistical model (Dias et al., 2018).

It’s considered that there were few systematic reviews or meta-analyses have pooled data of randomized controlled trials (RCTs) of dementia patients covering all above aspects, especially the novel non-pharmacological treatment approaches. There was no evidence in the literature to prove which interventions is the best for improving the cognitive function of dementia patients. Therefore, this study provided an optimal evidence-based basis for selecting non-pharmacological treatment options for dementia patients by comparing the magnitude effects of different non-pharmacological treatments on dementia cognition through the frequentist model of network meta-analysis (network meta-analysis).

## 2. Materials and methods

This systematic review was performed according to the Cochrane Handbook for the Systematic Review of Interventions (Deeks et al., 2019) and according to the Preferred Reporting Items for Systematic Reviews and Meta-Analyses (PRISMA) statement (Liberati et al., 2009). The review protocol was registered with PROSPERO (CRD42022363746).

### 2.1. Literature search strategies

The computer searched PubMed, Embase, Cochrane Central Register of Controlled Trials (CENTRAL), China National Knowledge Infrastructure Database, Wan Fang Database, Chinese Biomedical Literature Database, Web of Science, and VIP Database from their inception to 1 August 2022, without language restrictions. This article used the search terms “Dementia” OR “Senile Paranoid Dementia” OR “Alzheimer’s Disease” OR “Vascular Dementia” OR “Mixed Dementia” combined with a list of all included non-pharmacological therapies. In addition, this study supplemented the relevant literatures through manual search to obtain some of the relevant literature from the review literature and references in the Meta-analysis or reviews in our specialty, which could reduce to some extent the omission of literature that met the inclusion criteria of this study.

### 2.2. Inclusion and exclusion criteria

To include the central relevant published studies, the inclusion criteria for this study were as follows: Firstly, participants were over 65 years old and diagnosed with dementia by clinical examination tools such as National Institute on Aging and Alzheimer’s Association (NIA-AA) guidelines (Hyman et al., 2012), Diagnostic and Statistical Manual of Mental Disorders (DSM-IV-TR) (Wakefield, 2016), Neurological and Communicative Disorders and Stroke-Alzheimer’s Disease and Related Disorders Association (NINCDS-ADRDA) criteria (McKhann et al., 1984). All patients had no other primary or secondary disease; Secondly, interventions including EE, PBM, CCT, ET, and CST; Thirdly, comparisons were focused on core treatments (EE, PBM, CCT, ET, and CST) vs. other types of NPTs or control groups (CON). Fourthly, outcome indicators Mini-mental State Examination (MMSE), Montreal Cognitive Assessment (MOCA), and Alzheimer’s disease assessment scale (ADAS-cog) for symptom assessment of dementia patients; Fifthly, the type of study was a published RCT; Exclusion criteria were as follows: Firstly, studies whose research object was confounded with other cognitive impairment-related disorders, such as Parkinson’s, mild cognitive impairment, etc.; Secondly, studies in which various types of cognitive interventions were used in combination with each other; Thirdly, studies in which all regionalized versions of the MMSE scale, such as the Korean-MMSE (K-MMSE), Hong Kong-MMSE (H-MMSE); Fourthly, conference papers, or papers presented in abstract only; Fifthly, studies for which data could not be extracted because of missing or incomplete data (Middelstädt et al., 2016; Berman et al., 2017); Sixthly, duplicate publications.

### 2.3. Literature selection and data extraction

The literature was screened by reading the title and abstract for initial screening. After excluding irrelevant literature, the full text was further read to exclude the literature that can’t get the full text or can’t meet the inclusion criteria. A uniform data extraction form was used to extract data from the included literature, which including the first author, year of publication, country, study population type, age, sample size, male or female ratio, interventions, duration, frequency, and mean and standard deviation (SD) of outcome indicators. The screening process was independently performed by two investigators, which was screened the literature to extract information and cross-checking, and they will consult a third to assist in judgment in case of disagreement.

### 2.4. Quality assessment

The risk of bias 2 (ROB 2) in the included literature was evaluated by two researchers independently using the RCT risk of bias two assessment tool (Sterne et al., 2019) recommended by the Cochrane Handbook for Systematic Reviews of Interventions (Higgins, 2019), which was consist of five aspects of the randomization process, deviation from intended interventions, missing outcome data, measurement of the outcome, and selection of the reported results. Each entry was evaluated by the “low risk of bias,” “high risk of bias,” or “unclear.” If there was a disagreement, it will be decided by the third party or agreed upon by mutual agreement.

### 2.5. Statistical analyses

This study began with a similarity hypothesis test to evaluate the clinical and methodological similarity of the included studies (Salanti et al., 2008). A frequency science perspective was used to calculate efficacy of each treatment modality. This article analyzed the pooled data and demographic characteristics of each study and quantitatively estimated the heterogeneity of studies with I^2^ statistics (Chen and Benedetti, 2017) (ranging from 0 to 100%, the higher the I^2^, the more significant the heterogeneity, of which 25, 50, and 75% were considered as mild, moderate and high heterogeneity, respectively). After the network meta-analysis was conducted, the funnel plots were used to evaluate obvious publication biases based on visual inspection. Notably, this review used a random-effects model rather than a fixed-effects model because it might be the most appropriate and conservative analysis of the between-study variance (Kanters, 2022).

The STATA 16.0 was used to construct a network plot and provide all existing relationships, with different treatments represented by other nodes. The direct comparisons of results represented by lines connecting the appropriate nodes. The overall inconsistency and node split analysis were used to determine the inconsistency between direct and indirect evidence estimates for each intervention comparison, which was usually shown as *p*. If the *p* exceeds 0.05, the consistency model was used, indicating that there is no significant inconsistency (Higgins et al., 2012). The above analyses were performed using the “net-meta” package and the “Rjags” R language (X64 version 4.1.3). The rank probabilities of each treatment were calculated using the *p*-score, which values ranged from 0 to 1, where larger values indicated better treatment efficacy (Rücker and Schwarzer, 2015).

## 3. Results

### 3.1. Search process

**FIGURE 1:**
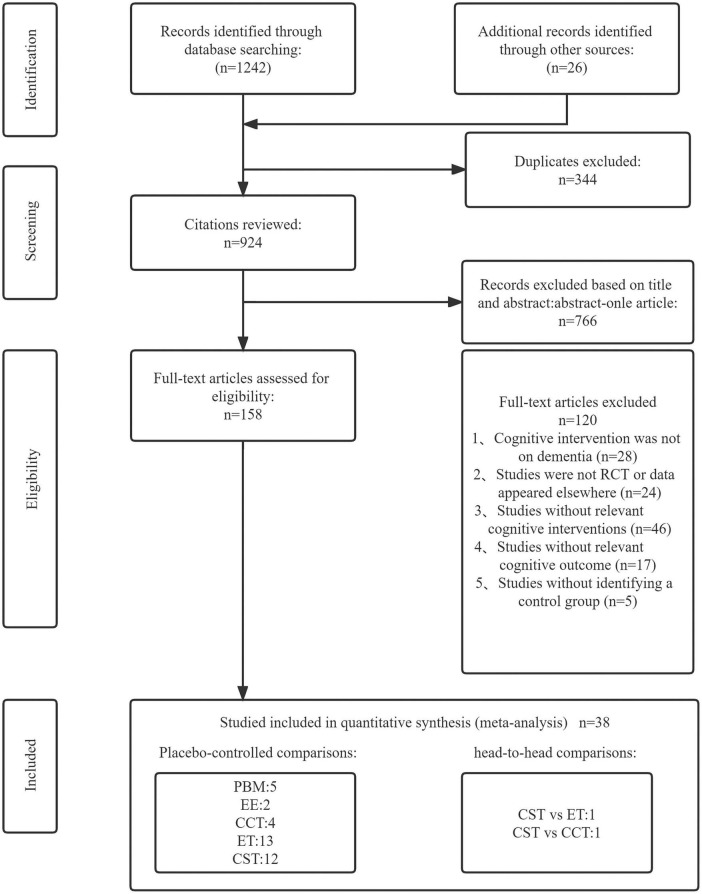
Literature review flowchart. EE, enriched environment; ET, exercise therapy; PBM, photobiomodulation; CCT, computerized cognitive training; CST, cognitive stimulation therapy; CON, control group.

The literature screening process was shown in Figure 1. The search for this study yielded a total of 1,268 articles. After removing 344 duplicates and 766 irrelevant articles, the remaining 158 articles were all read. Finally, 38 articles were included in our network meta-analysis by passing the strict eligibility criteria described above. All authors involved in this study agreed on the selection and evaluation method.

### 3.2. Baseline characteristics and ROB 2 quality assessment

Table 1 showed that baseline data on the demographic characteristics of the 38 included trials, which included 3,721 participants at baseline and 309 participants who did not complete the entire intervention, and leaved a total of 3,412 participants (experimental: 1,920, control: 1,942). The mean age of the subjects ranged from 70.04 ± 8.90 to 88.25 ± 5.15, and the duration ranged from 2 to 52 weeks, with a mean of 14 weeks. In addition, the mean MMSE scores of all selected studies ranged from 5.1 to 23.5. Meanwhile, among the 38 baseline data on demographic characteristics, three studies did not record baseline values of cognitive function in subjects (Cheung et al., 2019; Nagy et al., 2021; Rai et al., 2021), and three studies did not report exact baseline age data (Cavallo and Angilletta, 2019; Lok et al., 2020; Qi et al., 2021), but all conform to our inclusion criteria.

**TABLE 1:** Characteristics of included comparative studies.

References	Country	Type	Diagnose	Age (Mean ± SD)		Sample (Men)		MMSE (Baseline)		Duration (Weeks)	Frequence (d/W)	Time (min)
				NPT	CON	NPT	CON	NPT	CON			
Qi et al. (2021)	USA	PBM vs. CON	AD	NA		39 (23)	16 (8)	22.9 ± 2.4	23.2 ± 1.7	8	10	6
Chao (2019)	USA	PBM vs. CON	Dementia	80.5 ± 6.5	70.9 ± 5.9	4 (1)	4 (2)	19.5 ± 7.0	22.3 ± 1.3	12	3	20
Nizamutdinov et al. (2021)	USA	PBM vs. CON	Dementia	72.4 ± 8.2	77.8 ± 5.2	30 (17)	30 (16)	22.8 ± 2.6	23.2 ± 1.6	8	7	6
Kheradmand et al. (2022)	Iran	PBM vs. CON	Dementia	78.1 ± 6.19	76.1 ± 7.5	16 (7)	16 (7)	16.0 ± 6.9	15.1 ± 5.8	2	3	10
Nagy et al. (2021)	Egypt	PBM vs. CON	AD	69.5 ± 2.0	70.0 ± 2.0	30 (15)	30 (15)	NA		12	6	30
Zou (2017)	China	EE vs. CON	AD	72.8 ± 2.1	71.3 ± 3.5	37 (19)	18 (9)	13.0 ± 2.4	12.8 ± 1.2	12	5	30
Xu Ying et al. (2017)	China	EE vs. CON	AD	79.05 ± 9.5	78.9 ± 9.8	42 (19)	42 (20)	19.4 ± 5.1	18.2 ± 5.3	24	5	60
Oliveira et al. (2021)	Switzerland	CCT vs. CON	AD	All: 83.2 ± 5.7		10 (3)	7 (2)	18.6 ± 6.5	13.0 ± 7.5	8	2	45
Rai et al. (2021)	UK	CCT vs. CON	Dementia	74.0 ± 6.8	71.8 ± 8.5	31 (22)	30 (20)	NA		11	3	30
Lee et al. (2013)	Hong Kong	CCT vs. CON	AD	All: 77.7 ± 6.07		7 (1)	6 (2)	17 ± 3.58	15.3 ± 2.8	6	2	30
Cavallo and Angilletta (2019)	Italy	CCT vs. CON	AD	NA		36	36	22.7 ± 1.7	23.1 ± 2.4	12	3	30
Tárraga et al. (2006)	Spain	CCT vs. CST vs. CON	AD	CCT: 75.8 ± 5.9 CST: 77.4 ± 4.7	76.9 ± 4.5	CCT: 15 (5) CST: 16 (2)	12 (0)	CCT: 20.6 ± 2.1 CST: 22.5 ± 2.9	22.8 ± 2.4	24	3	20
Lamb et al. (2018)	UK	PT vs. CON	Dementia	All: 77 ± 7.9		329	165	22.0 ± 4.7	21.6 ± 4.6	28	2	60–90
Henskens et al. (2018)	Netherlands	PT vs. CON	Dementia	85.1 ± 4.6	84.7 ± 4.6	22 (5)	22 (5)	12.1 ± 6.4	10.2 ± 5.7	24	3	30–45
Toots et al. (2017)	Switzerland	PT vs. CON	Dementia	84.4 ± 6.2	85.9 ± 7.8	93 (23)	93 (22)	15.4 ± 3.4	14.4 ± 3.5	16	5	45
Telenius et al. (2015b)	Norway	PT vs. CON	Dementia	87.3 ± 7.0	86.5 ± 7.7	87 (24)	83 (21)	15.5 ± 0.6	15.7 ± 4.9	12	2	50–60
Yang et al. (2015)	China	PT vs. CON	AD	72.0 ± 6.7	71.9 ± 7.3	25 (10)	25 (7)	21.3 ± 2.2	20.0 ± 3.5	12	3	40
Aguiar et al. (2014)	Brazil	PT vs. CON	AD	78.6 ± 8.4	74.7 ± 7.4	17 (4)	17 (5)	20.1 ± 4.5	20.8 ± 4.0	24	2	40
de Oliveira Silva et al. (2019)	Brazil	PT vs. CON	AD	81.2 ± 8.9	77.5 ± 8.1	13 (8)	14 (3)	20.6 ± 4.9	21.4 ± 4.2	12	2	60
Öhman et al. (2016)	Finland	PT vs. CON	AD	78.0 ± 5.2	78.1 ± 5.3	140 (85)	70 (44)	18.2 ± 6.4	17.7 ± 6.2	48	2	60
Telenius et al. (2015a)	Norway	PT vs. CON	Dementia	86.9 ± 7.0	86.4 ± 7.8	82 (23)	81 (20)	15.6 ± 5.0	15.8 ± 5.0	14	2	50–60
Arcoverde et al. (2014)	Brazil	PT vs. CON	AD	78.5 ± 4.3	79 ± 1.9	10 (4)	10 (5)	20.4 ± 2.7	19.9 ± 3.4	12	2	30
Christofoletti et al. (2008)	Brazil	PT vs. CON	Dementia	72.9 ± 2.3	79.4 ± 2.0	17 (5)	20 (6)	12.7 ± 2.1	14.6 ± 1.2	24	3–5	60–120
Yan Lanyun et al. (2015)	China	PT vs. CON	AD	72.1 ± 6.1	70.6 ± 7.3	18 (8)	18 (7)	19.1 ± 3.1	20.6 ± 1.6	24	3	40
Wang et al. (2014)	China	PT vs. CON	AD	71.2 ± 7.0	70.0 ± 8.9	26 (12)	28 (9)	20.2 ± 3.6	19.4 ± 4.1	12	3	40
Spector et al. (2003)	British	CST vs. CON	Dementia	85.7 ± 6.2	84.7 ± 7.9	115 (24)	86 (19)	14.2 ± 3.9	14.8 ± 3.8	7	2	45
Orrell et al. (2014)	British	CST vs. CON	Dementia	82.7 ± 7.9	83.5 ± 7.2	123 (43)	113 (43)	17.8 ± 5.6	17.8 ± 5.4	7	2	45
Lok et al. (2020)	Turkey	CST vs. CON	AD	NA		30 (14)	30 (16)	17.6 ± 4.1	16.5 ± 4.1	7	2	45
Cheung et al. (2019)	Hong Kong	CST vs. CON	Dementia	81.8 ± 7.41	85.3 ± 6.6	18 (5)	12 (3)	NA		8	8	45–60
Cheng et al. (2014)	Hong Kong	CST vs. PT vs. CON	Dementia	CST: 81.9 ± 6.2 PT: 81.8 ± 7.4	80.9 ± 7.2	CST: 36 (13) PT: 39 (14)	35 (12)	CST: 19.0 ± 3.2 PT: 18.7 ± 3.9	18.9 ± 4.1	12	3	60
Andersen et al. (2012)	Norway	CST vs. CON	AD	81.6 ± 6.7	84.9 ± 3.3	103 (44)	77 (23)	22.9 ± 4.6	23.5 ± 4.3	52	5	30
Yamanaka et al. (2013)	Japan	CST vs. CON	Dementia	84.1 ± 5.5	83.7 ± 6.4	26 (6)	30 (6)	17.0 ± 4.2	16.9 ± 4.2	7	2	45
Stemmer et al. (2019)	Germany	CST vs. CON	Dementia	70.8 ± 10.0	80.2 ± 8.9	33 (16)	33 (19)	19.3 ± 3.9	18.5 ± 3.8	24	1	30
Cove et al. (2014)	UK	CST vs. CON	Dementia	76.8 ± 6.6	77.8 ± 7.7	24 (15)	23 (10)	22.7 ± 3.8	22.9 ± 3.0	14	1	45
Capotosto et al. (2017)	Italy	CST vs. CON	Dementia	88.3 ± 5.2	86.5 ± 5.7	20 (5)	19 (7)	18.3 ± 3.1	18.2 ± 3.6	7	2	35
Gibbor et al. (2021)	UK	CST vs. CON	Dementia	86.2 ± 5.2	77.2 ± 12.4	17	16	20.9 ± 3.0	22.5 ± 4.0	7	2	45
Tsantali et al. (2017)	Greece	CST vs. CON	AD	73.3 ± 4.9	74.2 ± 5.6	17	21	22.5 ± 0.9	23.1 ± 1.4	16	1	90
Yang (2019)	China	CST vs. CON	AD	75.74 ± 5.9	75.2 ± 7.0	42 (22)	42 (23)	18.5 ± 2.0	18.2 ± 2.0	7	2	30–45

AD, Alzheimer’s disease; VD, vascular dementia; EE, enriched environment; PBM, photobiomodulation; PT, physical exercise; CCT, computerized cognitive training; CST, cognitive stimulation therapy; CON, control group; NPT, non-pharmacological therapies.

**FIGURE 2:**
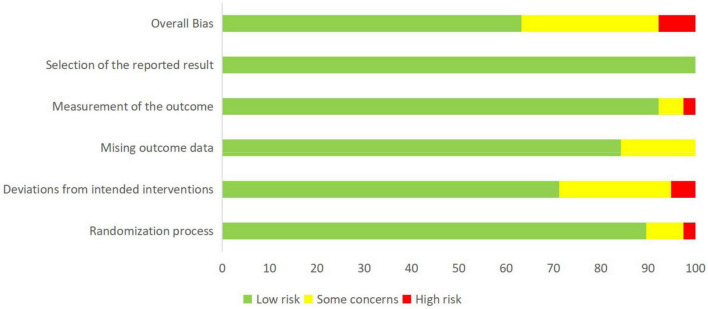
Summary of the risk of bias assessment of data included in network meta-analysis.

We evaluated the quality of included studies based on the Cochrane Collaboration Tool. Figure 2 and Supplementary Figure 1 summarized the risk of bias assessment for all data included in the network meta-analysis and the bias assessment risk of network meta-analysis at each outcome level in each study, respectively. We considered 63.2% as “low risk,” 7.9% as “high risk,” and 28.9% as “some concerns” about the articles of NPTs on cognitive function.

### 3.3. Network meta-analysis

The preliminary meta-analysis of the included studies showed mild heterogeneity (*I*^2^ = 64.3%). The symmetrical distribution of funnel plot indicated that there was no significant publication bias (*p* > 0.05) for Egger’s test, which showed that there was no significant bias in this study (Supplementary Figure 2).

**FIGURE 3:**
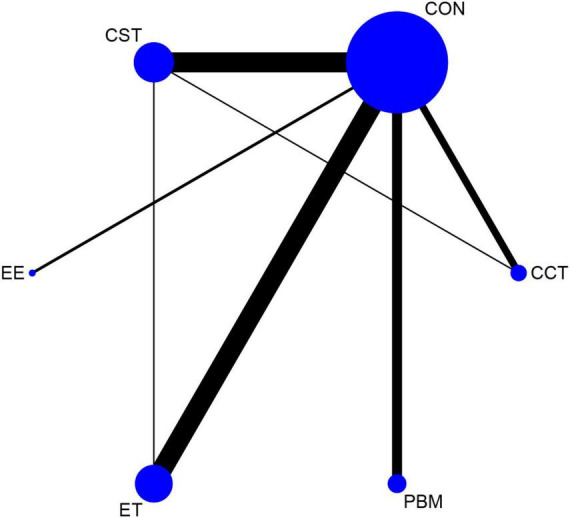
Comparison of cognitive interventions in a network meta-analysis.

The net evidence of different interventions was shown in Figure 3. A total of five interventions were included: EE, PBM, CCT, ET, and CST. According to the network plot, ET had the most studies, and CST had more studies, and EE had the least. CST and CCT formed a closed loop, as well as CST and ET also created a closed loop, which indicated both direct and indirect comparisons. There was no evidence of direct comparisons for the other interventions.

This article used global inconsistency and nodal splitting to test the inconsistency between direct and indirect evidence from the included studies, with *p* = 0.2859 for the former and results for the latter *p* = 0.7339 (CCT vs. CON), *p* = 0.3197 (CCT vs. CST), *p* = 0.1603 (CST vs. CON), *p* = 0.2735 (ET vs. CON), and *p* = 0.7978 (ET vs. CST) showed that none of the inconsistencies in evidence between direct comparisons for each cognitive intervention were statistically significant, indicating a good fit for consistency (Supplementary Figure 3).

**FIGURE 4:**
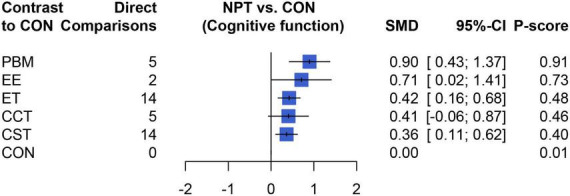
The forest plot of the effect of NPTs on cognition function.

Table 2 showed the results of the network meta-analysis of the primary outcomes, In terms of curative effect, most of the included NPTS were statistically significantly superior to the CON group, including PBM (SMD = 0.90, 95% CI: 0.43–1.37), EE (SMD = 0.71, 95% CI: 0.02–1.41), ET (SMD = 0.42, 95% CI: 0.16–0.68) and CST (SMD = 0.36, 95% CI: 0.11–0.62). Compared with the control group, the results of the CCT group (SMD = 0.41, 95% CI: −0.07–0.88) failed to show significant efficacy compared to the control group (*P* > 0.05). In addition, the comparison between different NPTs showed that PBM had a better treatment effect than CST, but there was no significant difference among other NPTs. Figure 4 showed the therapeutic effect ranking of each NPTS that PBM (*P*-score = 0.90) ranked the highest in improving cognitive function of dementia patients, which were followed by EE (*P*-score = 0.73), ET (*P*-score = 0.48), CCT (*P*-score = 0.46), and CST (*P*-score = 0.40) ranked the lowest.

**TABLE 2:** The effect of each non-pharmacological therapy on cognition based on cognition examination.

PBM	EE	ET	CCT	CST	CON
0.19 (–0.65; 1.03)					
0.48 (–0.06; 1.02)	0.29 (–0.45; 1.03)				
0.49 (–0.17; 1.16)	0.30 (–0.49; 0.76)	0.02 (–0.52; 0.55)			
**0.54 (0.00; 1.08)**	0.35 (–0.53; 1.14)	0.06 (–0.29; 0.41)	0.04 (–0.47; 0.56)		
**0.90 (0.43; 1.37)**	**0.71 (0.02; 1.41)**	**0.42 (0.16; 0.68)**	0.41 (–0.06; –0.87)	**0.36 (0.11; 0.62)**	

EE, enriched environment; PBM, photobiomodulation; ET, exercise therapy; CCT, computerized cognitive training; CST, cognitive stimulation therapy; CON, control group. Bolded values means there are statistical difference between NPTs and CON.

## 4. Discussion

As far as we know, from the science, from the perspective of frequency science, especially when combining traditional and more recent NPTs, previous studies have not proposed which intervention is the best way to treat cognitive function of dementia patients. Therefore, direct and indirect evidence were used to evaluate the relative effectiveness of different NPTs in cognitive interventions of dementia patients. According to the available data summarized in this study, the efficacy of the five NPTs were ranked from good to bad were: PBM, EE, ET, CCT, and CST. PBM, EE, ET, and CST show significant differences compared to CON (*p* < 0.05).

This study analyzed the improvement of cognitive function in patients with dementia by non-pharmacological treatments, which was consistent with the findings of a previous network meta-analysis (Wang et al., 2020). All NPTs had great potential to improve cognitive performance in patients with dementia, while CST and ET were shown to be the most beneficial interventions. Based on this, this study included additional new non-pharmacological treatment modalities and further evaluated their therapeutic effects compared with traditional non-pharmacological treatments such as CCT, CST, and ET. It was found that CST and ET had better therapeutic effects in improving cognitive function in patients. Notably, we found that PBM performed best in treating cognitive dysfunction in dementia patients by adding the latest non-pharmacological treatment modalities through frequency science perspective.

Photobiomodulation refers to a type of light therapy that utilizes that visible or near-infrared (NIR) light (600–1,100 nm) from lasers or light-emitting diodes (LEDs) stimulates or modulates various cellular and biological processes (Salehpour et al., 2018; Glass, 2021). Although most of the studies included in this article used red light or NIR at wavelengths from 650 to 1,080 nm for intracranial and intranasal irradiation methods, the treatment regimen for PBM was still largely dependent on patient-physician preference. Our findings were consistent with those reported in a previous systematic review (Zhu et al., 2022) that PBM was effective as a novel therapeutic approach to improve the level of cognitive function in dementia patients. This positive effect of PBM relied on four potential mechanisms (Hamblin, 2016), the basic ones involving photon absorption in the mitochondria (cytochrome c oxidase) (Hennessy and Hamblin, 2017), terminal enzymes in the electron transport chain, triggering downstream molecular and biochemical pathways in the mitochondrial respiratory chain, and exerting therapeutic effects (Lane, 2006; Karu, 2008; Hamblin, 2018); and adjust regional cerebral blood flow to increase perfusion levels (Chao, 2019; Salehpour et al., 2020; Baik et al., 2021); and open the light-mediated of calcium channels (Jung et al., 2019; Kim et al., 2020) and promote the activation of signaling mediators and transcription factors (Wu et al., 2022). In addition, PBM can reduce Aβ production and plaque formation by shifting amyloid precursor protein (APP) to non-amyloidogenic pathways (Zhang et al., 2020). These specific mechanisms of PBM are effective in improving mitochondrial function and increasing oxygen activity and ATP production, inhibiting aspects such as the downregulation of inflammation through inhibition of the NF-κB pathway. These aspects have a more significant role in improving cognitive function in patients with dementia. Moreover, the results of this study show that PBM has better efficacy in improving cognitive function methods in dementia patients compared to the other four non-pharmacological treatments. PBM therapy was a safe, non-invasive, non-thermal, and economical approach to improving cognitive function in patients with dementia while significantly reducing the pain of treatment, the adverse effects of treatment, and the financial burden on the family. In conclusion, PBM was a promising non-pharmacological option associated with cognitive improvement in patients with dementia. However, the optimal treatment regimen for different dementia severity and other modifying factors needs to be clarified to provide more precise individualized treatment plans in the future.

The rank probability of efficacy indicated that EE ranked second in effectiveness among the five different non-pharmacological interventions. This finding was similar to previous reports that EE was effective in improving cognitive function in patients with dementia (Bourdon and Belmin, 2021; Cutuli et al., 2022). EE was a non-invasive treatment that provides plasticity to the brain by combining cognitive training, such as memory and thinking, with dynamic stimulation, such as color, sound, and light, in an enriched environment (Figuracion and Lewis, 2021). A large number of animal studies have demonstrated the superiority and effectiveness of “Enriched environments” in improving cognitive functions in the brain (Nakano et al., 2020; Cordier et al., 2021). However, the network meta-analysis showed that there was no significant difference between EE and other non-pharmacological treatments, which may be due to the different study methods in the data pool. In the network meta-analyses, various interventions had slight interactions in the adjusted pooling and data comparison. Currently, there were few studies in this area of EE, and it’s a need to use more rigorous designs, more standard protocols, and more extensive studies to evaluate the effects of EE on improving cognitive function in patients with dementia.

The results of this study suggested that exercise therapy significantly improved the cognitive function of patients, which was consistent with previous meta-analyses that exploring the effects of exercise on dementia patients (Jia et al., 2019; López-Ortiz et al., 2021; Huang et al., 2022). In recent years, ET had been widely used as a low-risk and low-cost non-pharmacological treatment for patients with dementia. A large number of RCTs had reported the positive effects of exercise on cognitive function in patients with dementia (Henskens et al., 2018; Lamb et al., 2018; de Oliveira Silva et al., 2019). Exercise therapy improved cognitive performance mechanisms, such as increasing growth factors (Ruiz-González et al., 2021; Xue et al., 2022), modulating inflammatory cytokines (Hashiguchi et al., 2020; de Farias et al., 2021), alleviating oxidative stress (Hu et al., 2022), increasing cerebral blood flow (Lu et al., 2019; Tomoto et al., 2021), decreasing antibody concentrations (Giménez-Llort et al., 2013), and inhibiting tau phosphorylation from slowing the progression of dementia (Wang et al., 2021; Xu et al., 2022). However, these potential mechanisms have been proved to exist only in animal models, and some studies examining these mechanisms have not yet to prove their applicability to humans. This research included medium and long-term aerobic exercise, resistance training, physical and mental exercise, Tai Chi exercise and multi-component exercise. There was moderate heterogeneity among all RCTs on ET, which indicated that ET may have some variability in cognitive improvement due to the differences in exercise modality, intensity, frequency, and duration of study design. It was worth noting that recent studies had some differences in exploring the effects of different exercise modalities on cognitive improvement. Some studies have found that multi-component exercise could better improve cognitive dysfunction in patients (McDermott et al., 2019; Yang et al., 2021). Recent studies showed that resistance training appears to have the best therapeutic effect on improving cognitive function in patients (Huang et al., 2022). Therefore, future research on the process of exercise therapy for cognitive improvement needs to describe more specific exercise modalities and find more accurate ways to mitigate the process of dementia.

The results showed that there was no significant difference in the effect of CCT on the cognitive performance of patients with dementia, which was consistent with the findings of previous meta-analysis studies (Gates et al., 2019). Several studies found that cognitive training improves cognitive function in multiple cognitive domains in patients with mild cognitive impairment and dementia (Kanaan et al., 2014; Trebbastoni et al., 2018). CCT, a new cognitive training system that presents cognitive training tasks in a computer program, had a better effect on cognitive function (Bauer and Andringa, 2020). Despite the vital role of CCT in improving cognitive function, the current findings were not optimistic, which may be due to fewer included studies or the lower sensitivity of MMSE to cognitive function changes than other scales (Fu et al., 2017). All RCTs in this study used the MMSE as an assessment tool, which can’t accurately evaluates subtle changes in cognitive function (Weuve et al., 2006).

Cognitive stimulation therapy can improve cognition more effectively than controls, which was consistent with the previous meta-analysis that reported a more significant effect on cognitive function (Saragih et al., 2022). Using repetitive activities, especially tasks and games can help improve brain connectivity and generate new synapses and myelinated neural circuits, which was contributed to restoring or reorganizing neuronal structures behind cognitive function (Bryck and Fisher, 2012). Meanwhile, studies showed that CST has better clinical efficacy than drug therapy (Liang, 2019; Devita et al., 2021). It was noteworthy that CST did not show better efficacy compared to other non-pharmacological therapies in the present study.

Our study also had some limitations. First, the quality of the included studies was moderately heterogeneous due to the significant differences in treatment frequency, and treatment modality between different NPTs. Secondly, EE in our network only included a few studies, and there was less evidence-based evidence from studies of EE as a new non-pharmacological treatment modality, which may make the results biased. Thirdly, most of the included studies in this network meta-analysis compared non-pharmacological treatments with controls, while the number of actual head-to-head trials was relatively small, so comparative efficacy between interventions was often based on indirect comparisons. Fourthly, although we assessed the three assumptions of the network meta-analysis (homogeneity assumption, transferability assumption, and consistency assumption) to ensure their plausibility, there was moderate heterogeneity. Finally, our study did not analyze the safety of cognitive interventions because only four included studies described their adverse effects.

## 5. Conclusion

In conclusion, our network meta-analysis concluded that the best non-pharmacological treatment modality for patients with dementia was PBM, followed by EE, ET, and CST. However, the results should be interpreted with caution, considering the limitations of our meta-analysis described above and the insufficient number of studies in the existing literature. In the future, more multi-arm randomized controlled trials should be conducted to provide more direct evidence for the relative effectiveness of various non-pharmacological treatment.

## Data availability statement

The original contributions presented in this study are included in the article/Supplementary material, further inquiries can be directed to the corresponding author.

## Author contributions

FG played a guiding role in the manuscript writing and revision and data analysis. FW contributed to the conception or design of the work, the writing and revision of the manuscript, and the processing of the data. All authors contributed to the article and approved the submitted version.
