# Supplementary material for: Effectiveness of non-pharmacological therapies on cognitive function in patients with dementia—A network meta-analysis of randomized controlled trials
Source: Front Aging Neurosci. 2023 Mar 2;15:1131744. doi: 10.3389/fnagi.2023.1131744 (PMC10035791; doi:10.3389/fnagi.2023.1131744)
Supplement: Supplementary file 1 [file Data_Sheet_1.docx]

Supplementary Material

**Effectiveness of non-pharmacological therapies on cognitive function in patients with dementia——A network meta-analysis of randomized controlled trials**

Guangxin Luo^1^, Junqiu Zhang^1^, Zeyi Song^2,^, Ying Wang^1^, Xiaojing Wang^1^, Haifeng Qu^2^, Chengjiang Liu^3^, Fujia Gao^1*^

*** Correspondence:** Fujia Gao: gaofujia1983@163.com

# Supplementary Figures

**Supplementary Figure S1.** Risk of bias assessment within the individual studies at outcome level

**
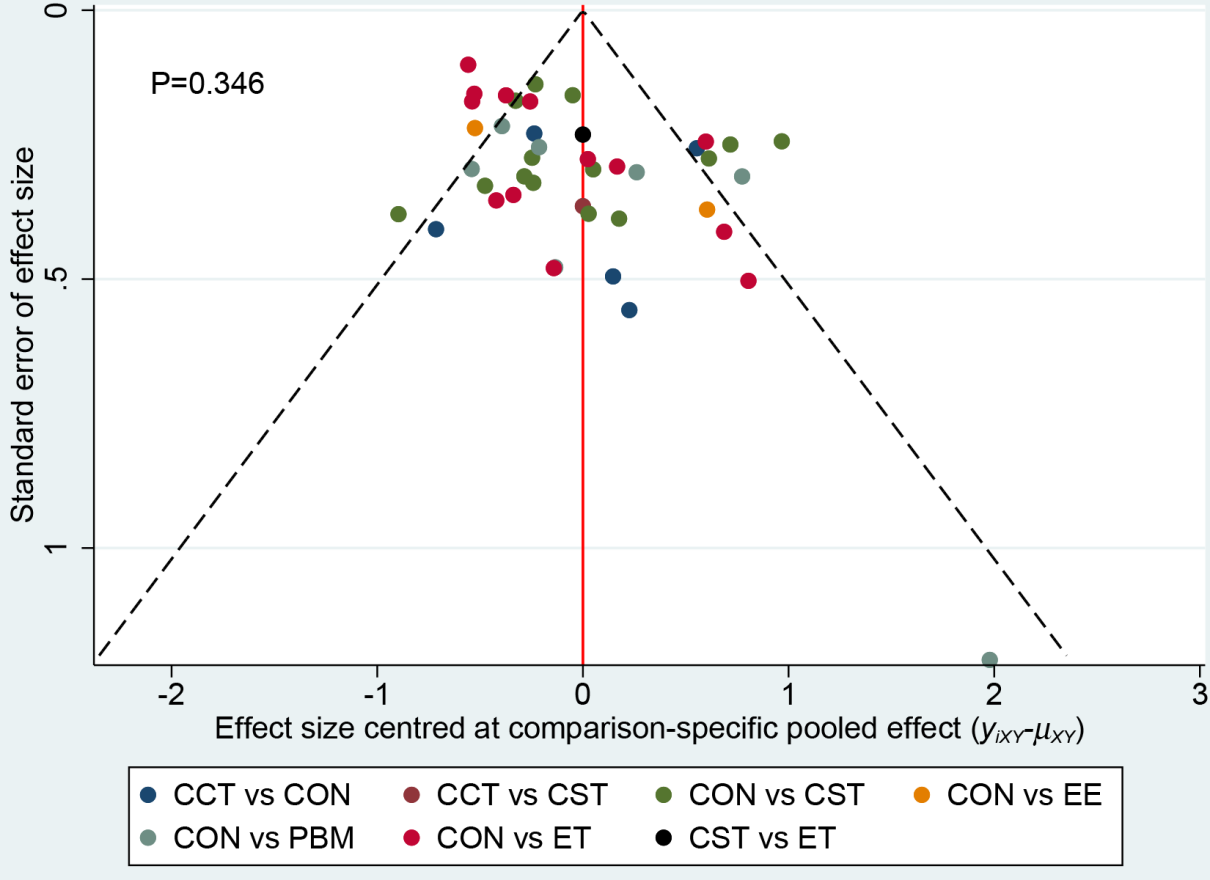
Supplementary Figure S2.** Network funnel plot of included studies.


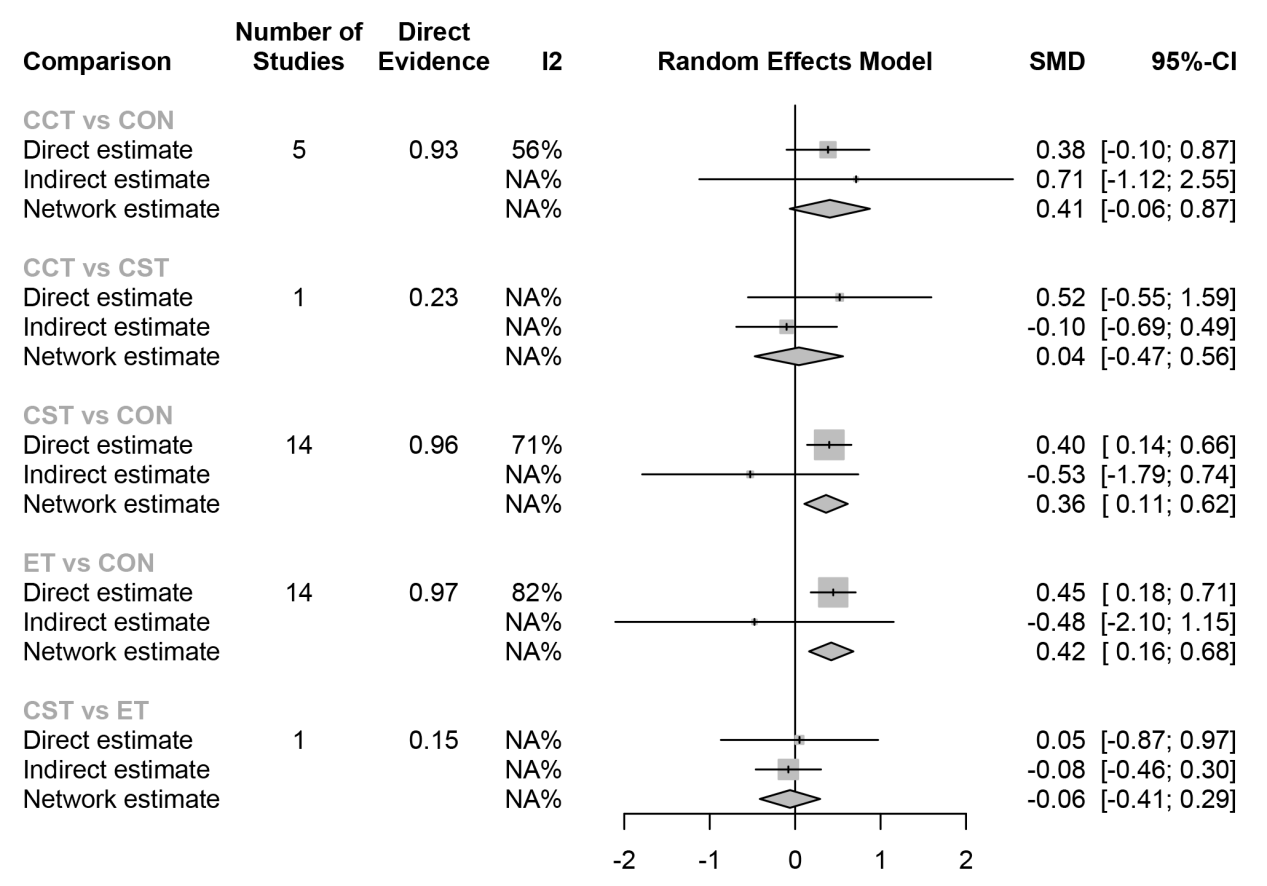
**Supplementary Figure S3.** Network incoherence between direct and indirect estimates for the outcome.
